# Improvement of the YOLOv5 Model in the Optimization of the Brown Spot Disease Recognition Algorithm of Kidney Bean

**DOI:** 10.3390/plants12213765

**Published:** 2023-11-03

**Authors:** Pengyan Su, Hao Li, Xiaoyun Wang, Qianyu Wang, Bokun Hao, Meichen Feng, Xinkai Sun, Zhongyu Yang, Binghan Jing, Chao Wang, Mingxing Qin, Xiaoyan Song, Lujie Xiao, Jingjing Sun, Meijun Zhang, Wude Yang

**Affiliations:** 1 College of Agriculture, Shanxi Agricultural University, Taigu, Jingzhong 030801, China; sz15003479431@163.com (P.S.); wxy728111@126.com (X.W.); sjj821202@163.com (J.S.);; 2 College of Resources and Environment, Shanxi Agricultural University, Taigu, Jingzhong 030801, China

**Keywords:** kidney bean brown spot, disease detection, YOLOv5-SE-BiFPN, feature fusion, attention mechanism

## Abstract

The kidney bean is an important cash crop whose growth and yield are severely affected by brown spot disease. Traditional target detection models cannot effectively screen out key features, resulting in model overfitting and weak generalization ability. In this study, a Bi-Directional Feature Pyramid Network (BiFPN) and Squeeze and Excitation (SE) module were added to a YOLOv5 model to improve the multi-scale feature fusion and key feature extraction abilities of the improved model. The results show that the BiFPN and SE modules show higher heat in the target location region and pay less attention to irrelevant environmental information in the non-target region. The detection Precision, Recall, and mean average Precision (mAP@0.5) of the improved YOLOv5 model are 94.7%, 88.2%, and 92.5%, respectively, which are 4.9% higher in Precision, 0.5% higher in Recall, and 25.6% higher in the mean average Precision compared to the original YOLOv5 model. Compared with the YOLOv5-SE, YOLOv5-BiFPN, FasterR-CNN, and EfficientDet models, detection Precision improved by 1.8%, 3.0%, 9.4%, and 9.5%, respectively. Moreover, the rate of missed and wrong detection in the improved YOLOv5 model is only 8.16%. Therefore, the YOLOv5-SE-BiFPN model can more effectively detect the brown spot area of kidney beans.

## 1. Introduction

Kidney bean is an important economic crop with very high nutritional value [1]. During the growth process, kidney beans are susceptible to a variety of pests and diseases, among which brown spot disease is relatively common, which can cause drying and shedding of plant leaves, affect the pod formation of plants, and ultimately lead to yield loss. Timely monitoring and control measures can prevent the further spread of brown spot disease in kidney beans, thus reducing the damage to plants and ensuring the healthy growth of kidney beans [2]. Traditional methods of monitoring brown spot disease in kidney beans are mainly based on observing the color, size, and shape of spots on kidney bean leaves by manual means such as visual observation, field inspections, and sample collection to judge the severity of the disease [3]. However, these methods generally have the disadvantage of being more subjective, time-consuming, inefficient, and difficult to achieve in real-time monitoring [4]. In recent years, with the rapid development of computer technology, an increasing number of scholars have utilized techniques such as computer vision and machine learning to identify crop leaf features for rapid detection and classification of different crop diseases [5,6,7,8,9]. Poornima et al. [10] utilized image processing and machine learning techniques to analyze and detect plant diseases and achieved early and periodic detection. Khalid et al. [11] detected diseases on plant leaves in real-time by using the YOLOv5 model, and the accuracy reached more than 93%. Bansal et al. [12] used a Convolutional Neural Network (CNN) to classify apple diseases, and the model classifier’s accuracy reached 90%. Mathew et al. [13] used YOLOv5 to detect bacterial mottle disease on pepper leaves and provided farmers with an effective disease monitoring and control method by analyzing and identifying diseases in real-time in a farm environment. It could be seen that the application of deep learning technology provides an efficient and accurate solution for plant disease detection, which is of great significance for the timely detection of diseases and taking corresponding measures to promote the sustainable development of agriculture [14].

The YOLOv5 [15] model is a deep learning-based target detection model, which is one of the preferred models in the field of target detection because its main feature is that it significantly improves the detection speed while maintaining high detection accuracy. However, in tasks dealing with specific crop disease recognition, the model is difficult to accurately select and focus on the important features of crop diseases and is prone to overfitting phenomena, which leads to low model recognition efficiency. To overcome these challenges and further enhance the accuracy and efficiency of kidney bean disease detection, this study introduces two key techniques: the Bi-Directional Feature Pyramid Network (BiFPN) and Squeeze-and-Excitation (SE) modules.

Bi-Directional Feature Pyramid Network (BiFPN) [16] is an efficient feature fusion network structure for target detection and semantic segmentation that is widely used in image processing and computer vision tasks. Through a bidirectional feature propagation mechanism, multi-layer features are integrated and utilized to better capture the multi-scale features of the target. For example, Kumar et al. [17] introduced BiFPN in the DenseNet-201 Backbone network to achieve bi-directional extraction of deep and shallow features and to guide the weight distribution to effectively optimize the output vector of the YOLOv5 network. Lin et al. [18] introduced BiFPN into the YOLO model to solve the problem of difficulty in recognizing tea diseases with small targets. Hu et al. [19] improved the effectiveness of the model in detecting insect pests by replacing the PANet with the BiFPN network. The addition of BiFPN to the YOLOv5 structure enables the model to better capture features at different scales and improves the model’s sensory field and semantic information expression, resulting in a significant improvement in the detection of crop pests and diseases. The development of attention mechanisms was inspired by the imitation of human vision [20]. The human visual system has demonstrated superior ability in processing complex images and extracting key features from complex backgrounds. Therefore, researchers applied this visual attention mechanism to machine vision [21], enabling the model to automatically select and attend to the parts of the input data that are more important to the task, thus improving the model’s performance. Soft attention can be more conveniently used in deep learning to compute attention weights iteratively, and thus it has been widely used in related research. Hu et al. [22] proposed a Squeeze and Excitation (SE) attention network, which extracts key features by weighting each feature channel. By adding SE to the YOLOv5x model, Zhang et al. [23] enabled the model to show the highest performance in weed and lettuce plant identification. The SE module effectively extracts key features of tomato virus disease by weighting each feature channel, which enables the network to pay more attention to the important feature channels and at the same time reduces attention to irrelevant information, which improves the model’s ability to recognize key features.

In this study, we improved the existing disease detection model by introducing the BiFPN structure and SE module, highlighting the local features of the recognition object, and filtering the key features so that the model focuses on the accurate recognition of the disease region while maintaining the detection speed of the original YOLOv5 model, to improve the ability of filtering the key features of the brown spot disease of kidney bean and to further optimize the detection performance of the model for brown spot disease of kidney bean.

## 2. Results and Discussion

### 2.1. Validation of BiFPN and SE Module Effects

**Figure 1:**
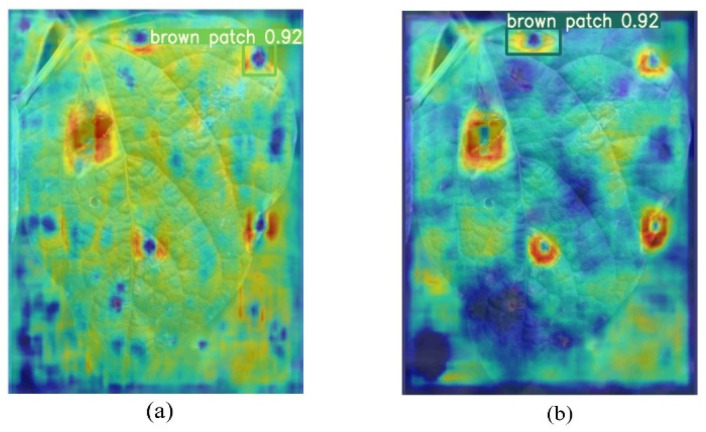
Comparison of the effectiveness of PANet and BiFPN-SE in recognizing brown spot heat maps. (**a**) Structural heat map for PANet, (**b**) Structural heat map for BiFPN-SE.2.2. Comparison of Model Evaluation Indicators.

Grad-CAM helps to understand the focus of the model by generating heat maps that indicate the regions of interest of the model in the input image [24]. To more accurately assess the contribution of BiFPN-SE in the network, this study compares the visualization effects of PANet and BiFPN-SE structure-based networks using the Grad-CAM technique [25,26,27]. Through the weighted fusion of the BiFPN structure and other feature layers, the attention of the target region is precisely adjusted [28], while the influence of irrelevant features is attenuated by the SE-adaptive Squeeze and Excitation operations for each feature channel, so that the network better understands the context and details of the target [29], and the hotspot location shown in the final heatmap is the same as that of the actual target as shown in Figure 1b. Compared to the PANet structure of YOLOv5 (Figure 1a), the network of BiFPN-SE exhibits higher thermal power in the target location region while paying less attention to irrelevant environmental information in non-target regions.

#### 2.1.1. Ablation Studies

In this study, the YOLOv5, YOLOv5-SE, YOLOv5-BiFPN, and YOLOv5-SE-BiFPN models were evaluated using the same dataset, and the results were compared with the YOLOv5-SE-BiFPN model, all under the same optimization parameters (Table 1). Comparative test results revealed that the YOLOv5-SE-BiFPN model achieved significantly higher recognition Precision, with an increase of 4.9%, 1.8%, and 3%, respectively, compared to the other three models. The YOLOv5-SE and YOLOv5-BiFPN models performed better relative to the basic YOLOv5 model, and YOLOv5-SE could better capture the features of brown spot disease through adaptive enhancement of essential feature channels and feature optimization. Additionally, the YOLOv5-BiFPN model provided richer contextual information through multi-scale feature fusion, enabling more accurate detection and localization of brown spot disease of various sizes and shapes. By comparing the experimental results with the relevant literature [30,31], it was found that combining BiFPN with SE enabled the YOLOv5-SE-BiFPN model to achieve a more significant performance improvement in the identification of brown spot disease in kidney beans. 

**Figure 2:**
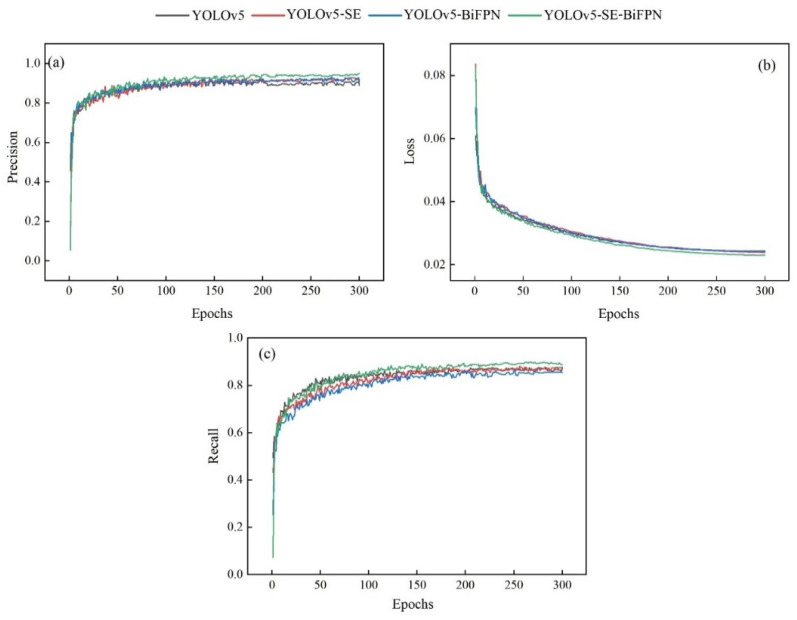
The curve of change of the model evaluation index: (**a**) Precision, (**b**) Loss function, and (**c**) Recall.

Figure 2 shows the variation curves of Precision, Loss, and Recall for the four models.

According to Figure 2a,c, after 100 epochs, the curves of the other three models except YOLOv5 were in the stage of slowly rising. Then, after 150 epochs, the curves of the YOLOv5-SE and YOLOv5-BiFPN models tended to flatten out, and the curve of the YOLOv5-SE-BiFPN model continued to rise, exhibiting small fluctuations. This indicates that the YOLOv5-SE-BiFPN model has higher detection Precision and has maintained stable improvement in its ability to recognize true positive examples across all thresholds. In Figure 2b, all four models’ curves showed a slow decreasing trend, but the YOLOv5-SE-BiFPN model’s curve consistently remained below the other models’ curves. This indicates that the four models gradually adapted to the sample data during the training process, while the YOLOv5-SE-BiFPN model, with relatively low loss values, was able to converge more quickly during training and achieve better performance at the end of training.

#### 2.1.2. Comparison of Classical Target Detection Models

To evaluate the performance of the YOLOv5-SE-BiFPN model, it is compared with the faster R-CNN and EfficientDet models, which are classical in the field of target detection. By calculating the Precision, Recall, and mAP of the three models, the results are presented in Table 2. The Precision of the YOLOv5-SE-BiFPN model was 94.7%, which was improved by 9.4% and 9.5% compared with the Faster R-CNN and EfficientDet models, respectively. Furthermore, the Recall of the YOLOv5-SE-BiFPN model was 88.2%, mAP@0.5 was 92.5%, and mAP@0.5:0.95 was 69.5%. Compared with the other two models, the YOLOv5-SE-BiFPN model exhibited higher performance in all metrics. Combining the experimental results with the Fast R-CNN and EfficientDet models for disease recognition studies [32,33], it was found that the YOLOv5-SE-BiFPN model performs better in the detection of brown spot disease in kidney beans and can more accurately detect and localize the disease area. 

**Figure 3:**
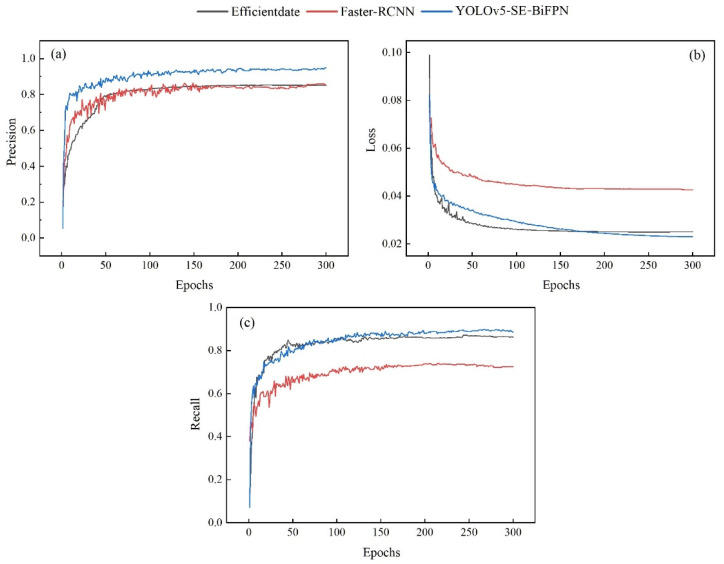
The curve of change of the model evaluation index: (**a**) Precision, (**b**) Loss function, and (**c**) Recall.

The changing curves of Precision, Loss, and Recall of the three models were compared and analyzed, and the corresponding change curves were plotted (Figure 3). From Figure 3a, it can be seen that during the training process, the Precision of all three models showed a gradual upward trend with the increase in the number of iterations. Compared with the other two models, the characteristic curve of the YOLOv5-SE-BiFPN model rose obviously and smoothly, and the Precision rose the fastest, with less fluctuation at convergence. In Figure 3b, it can be seen that the loss function value of the YOLOv5-SE-BiFPN model decreased faster, which meant that the model could converge quickly and learn the features in the data effectively. When comparing the Recall curves of the three models (Figure 3c), the YOLOv5-SE-BiFPN model and the EfficientDet model had a similar probability of correct disease detection, but the former was slightly higher, and the changing curve of the model was more pronounced and smoother, suggesting that the model correctly detected more areas of brown spot disease.

In summary, compared with the Faster R-CNN and EfficientDet models, the YOLOv5-SE-BiFPN model performed better in terms of detection performance and recognition of brown spot disease in kidney beans. 

### 2.2. Comparison of Model Prediction Results

**Figure 4:**
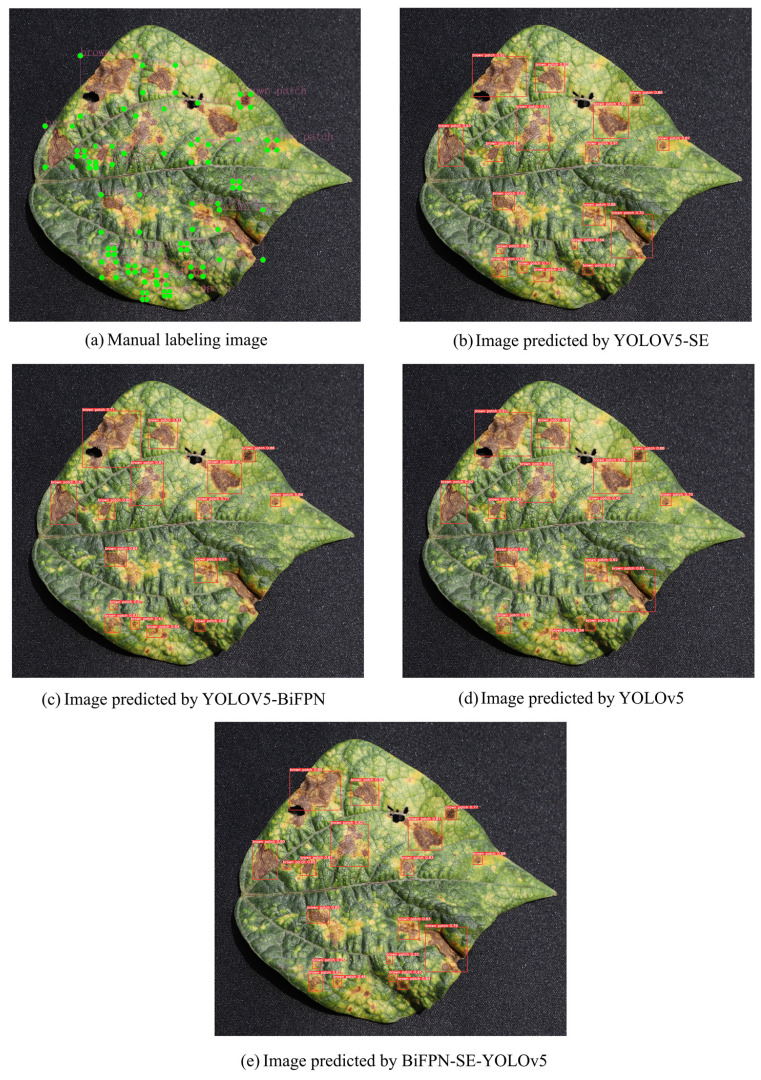
Comparison of manual labeling and results of model prediction.

YOLOv5, YOLOv5-SE, YOLOv5-BiFPN, and YOLOv5-SE-BiFPN were evaluated on the test set, and the prediction results are shown in Table 3. The accuracy of the YOLOv5-BiFPN model had been increased by 4.77%, 0.16%, and 0.7%, which further proved the accuracy of the model in detecting brown spots in kidney beans, and some of the results are shown in Figure 4.

Figure 4a shows the 24 manually labeled disease areas, and Figure 4b–e shows the prediction results of the YOLOv5-SE, YOLOv5-BiFPN, YOLOv5, and YOLOv5-SE-BiFPN models, respectively. Among them, the two models, YOLOv5 and YOLOv5-BiFPN, had a higher rate of missed and wrong detections, respectively (Table 3). By contrast, the YOLOv5-SE-BiFPN model, which only has a rate of missed and wrong detections of 8.16%, demonstrated stronger generalization ability. The introduction of the attention mechanism and the weighted feature fusion structure suppressed the key feature channels by filtering the useless or less contributing features, thus increasing the contrast between the lesion area and the background and reducing the error rate of detection.

### 2.3. Comparison of Model Feature Visualization

**Figure 5:**
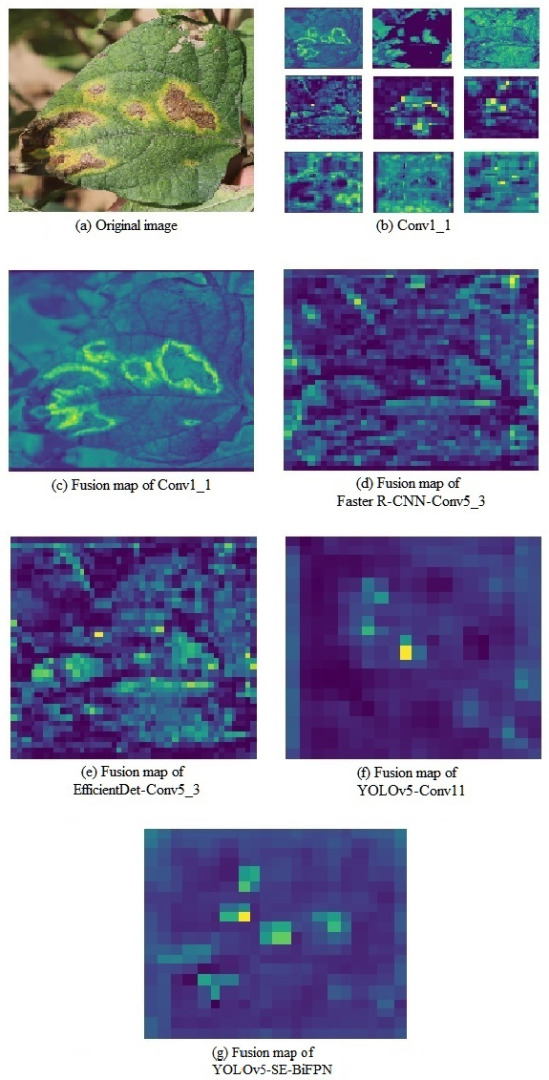
Visualization of the feature map.

To further analyze the performance of the model, in this study, some of the feature extraction layers were visualized, and the feature maps of all the channels in this feature extraction layer were overlaid and merged (Figure 5). Figure 5a shows the original image; Figure 5b shows the feature maps of the nine convolutional kernels in the shallow convolutional layer; Figure 5c shows the overall feature maps of all the channels in this layer after 1:1 fusion; and Figure 5d–g shows the overall feature maps of the deep convolutional layers of the Faster R-CNN, EfficientDet, YOLOv5, and YOLOv5-SE-BiFPN models, respectively.

By contrasting Figure 5f,g, it was found that there were three highlighted regions in Figure 5f and six highlighted regions in Figure 5g, which meant that there were three and six kidney bean brown spot disease regions, respectively, whereas there were six brown spot disease regions in the original image. This result indicated that the improved YOLOv5-SE-BiFPN model judged the disease regions more accurately, and it was mainly attributed to the fact that the YOLOv5-SE-BiFPN model’s deep network layer effectively improved the detection Precision of the model by filtering the extracted features and introducing the SE module to weight the key features so that the model paid more attention to the key features.

## 3. Materials and Methods

### 3.1. Image Acquisition

**Figure 6:**
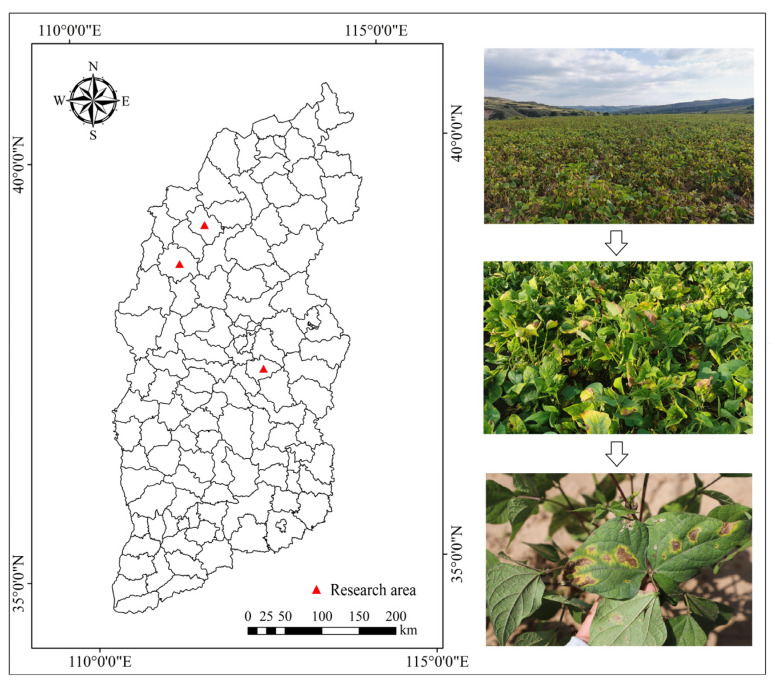
Schematic diagram of the sampling area.

Data for this study were collected from the test field in Taigu District, Jinzhong City, Shanxi Province, and the test fields in Kelan and Shenchi Counties, Xinzhou City, with the sampling areas shown in Figure 6. The collection time was from 19 June to 3 September 2022 and 2023. To ensure the quality of imaging, the collection time was uniformly specified as 11:00–14:00, and a Canon 6D2 camera (26.2 megapixels) was used to take photos of the diseased leaves of kidney beans in the natural environment under sufficient outdoor natural light. A total of 1204 data samples of brown spot disease of kidney beans have been collected in the experiment and stored in JPG format.

### 3.2. Image Preprocessing

To exclude the interference of the surrounding environment and other leaves and to make sure that the model only focuses on single-leaf features, a cropping operation was performed on 1204 kidney bean disease samples, and each image was adjusted as a whole to contain only a single leaf to eliminate the interference of the background and the environment and to provide a reliable source of data for the subsequent analysis of disease detection [34].

**Figure 7:**
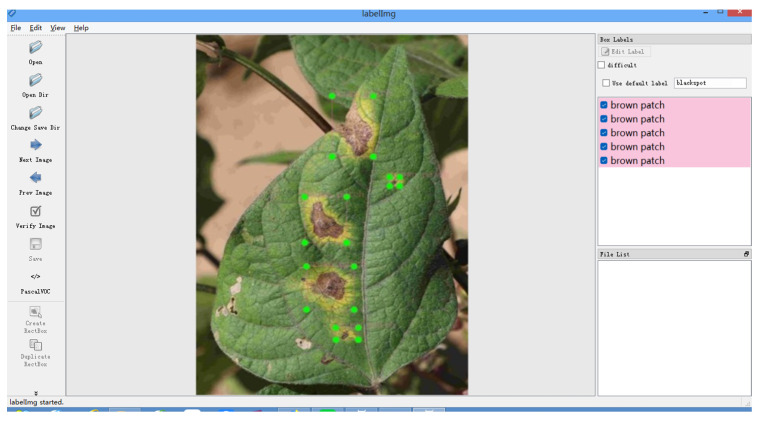
Data labeling.

Secondly, the experimental data were manually labeled using LabelImg1.8.6 [35]. According to the characteristics of the disease in a brown spot of kidney beans, the spot area was accurately located, the rectangular bounding box was drawn and named brown patch, and finally, the corresponding XML label file was generated, and the image labeling process was shown in Figure 7.

**Figure 8:**
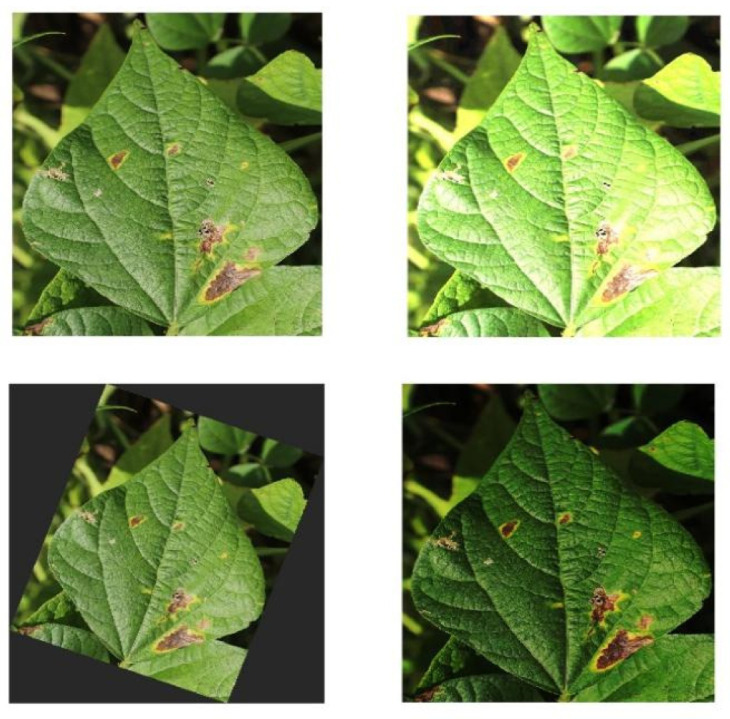
Data enhancement.

To increase the diversity of the training data, improve the generalization ability and robustness of the model, and avoid overfitting, data enhancement was performed on the original data in this study. The kidney bean brown spot disease dataset was expanded to 4989 images using random rotation, brightness variation, and so on (Figure 8). 

### 3.3. YOLOv5-SE-BiFPN Model Construction

#### 3.3.1. Bi-Directional Feature Pyramid Network (BiFPN)

**Figure 9:**
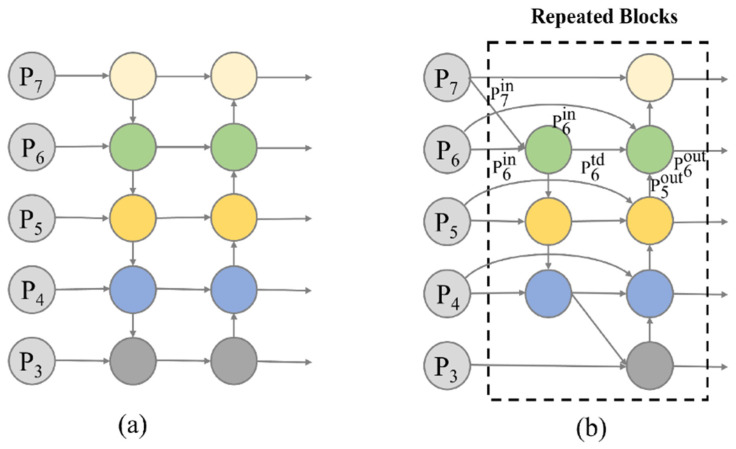
PANet and BiFPN network structures. (**a**) Structure of PANet (bottom-up secondary fusion) and (**b**) BiFPN (edges with contextual information added to the original FPN module), the circles with different colors in the figure represent different levels of features.

The neck layer of the YOLOv5s network utilizes the FPN + PANet structure, and the PANet structure adds a bottom-up interlayer transmission path to improve the connection between the high-level semantic information and the underlying positional information, enhancing the feature fusion capability of the neck (Figure 9a). However, when fusing input features of different resolutions, these features are simply summed and usually do not contribute equally to the fused output features. To address this problem, Tan et al. [36] proposed BiFPN, which is based on efficient bidirectional cross-scale connectivity and weighted multiscale feature fusion. BiFPN introduces learnable weights to learn the importance of the different input features while performing both top-down and bottom-up multiscale feature fusion (Figure 9b).

The BiFPN network ignores nodes with only one input edge. The network considers that if a node has only one input edge, it contributes little to the feature network because it does not have enough information for effective feature fusion and contributes less to the network. In addition, an extra edge is added between the input and output nodes in the same layer to obtain a higher level of fused features through iterative stacking. This strategy helps to improve the feature fusion efficiency and accuracy of the BiFPN network, which enhances the feature extraction ability of the model for a specific scene.

The BiFPN network aims to achieve an effective fusion of input features with different resolutions and consider their contributions to the output features. Firstly, the learning parameters are set to ensure the consistency of the input layer size and to add additional weights to each feature layer. Secondly, a fast normalized fusion method is used to weigh the feature layers. The fusion process is as shown in Equations (1)–(3), where Resize denotes upsampling or downsampling. The “w” denotes the weight parameter of the feature layer:

(1) $$O=\sum_{i}\frac{w_{i}\cdot I_{i}}{\epsilon +\sum_{j}w_{j}},$$

(2) $$P_{6}^{\mathrm{td}}=\mathrm{Conv}\left(\frac{w_{1}\cdot P_{6}^{\mathrm{in}}+w_{2}\cdot \mathrm{Resize}\left(P_{7}^{\mathrm{in}}\right)}{\epsilon +w_{1}+w_{2}}\right),$$

(3) $$P_{6}^{\mathrm{out}}=\mathrm{Conv}\left(\frac{w_{1}^{\prime }\cdot P_{6}^{\mathrm{td}}+w_{2}^{\prime }\cdot P_{6}^{\mathrm{td}}+w_{3}^{\prime }\cdot \mathrm{Resize}\left(P_{5}^{\mathrm{out}}\right)}{\epsilon +w_{1}^{\prime }+w_{2}^{\prime }+w_{3}^{\prime \prime }}\right).$$

#### 3.3.2. Squeeze-and-Excitation (SE) Module

**Figure 10:**
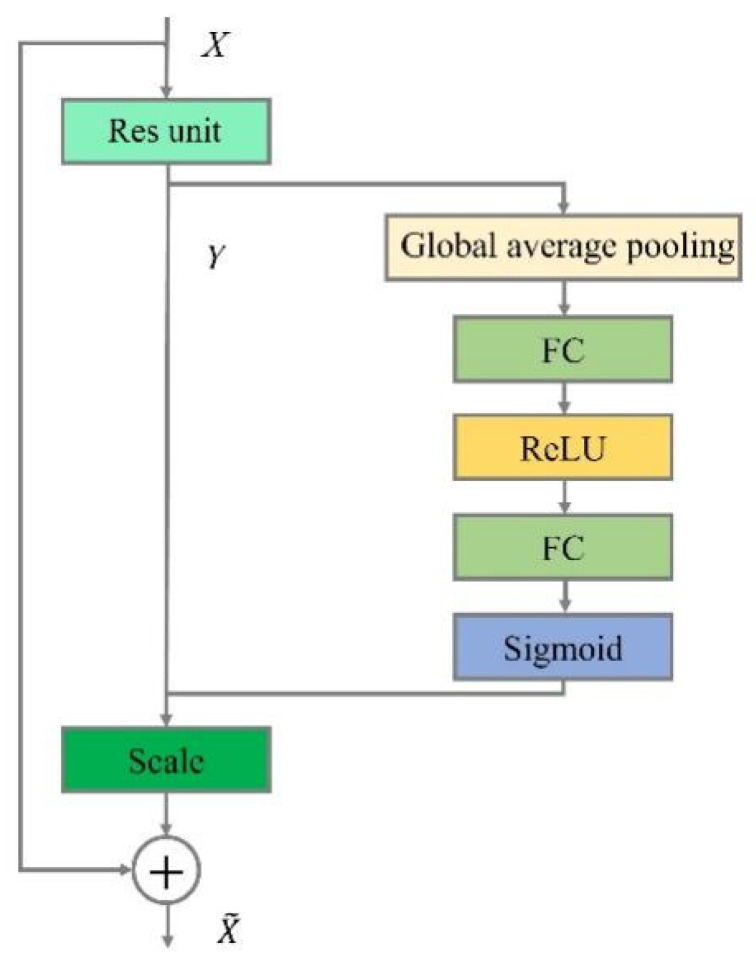
SE module.

In this study, the SE attention mechanism (Figure 10) is added to the Backbone network so that the model learns the weights of each image feature by increasing the weights of valid features and decreasing the weights of invalid or useless features, thus enabling the network model to be trained to produce the best results. The feature maps are first pooled globally on average, then the weights and biases of the channels are learned separately through two fully connected layers, and finally the weights of each channel are normalized to the range of [0, 1] using a sigmoid function. The SE module can adaptively adjust the weights of the different channels so that the network can better express the features of the images, and by weighting and merging the image features of the brown spot disease of the kidney bean, it can improve the performance of the network at a smaller computational cost.

The SE module is a computational block that can be used to convert the input feature vector X to the output feature mapping. The conversion relation is shown in Equation (4). Where * denotes the convolution operation and $v_{c}$ is the output feature mapping, $v_{c}=\left[v_{c}^{1},v_{c}^{2},...,v_{c}^{C^{\prime }}\right]$, X is the input feature vector, $X=\left[X^{1},X^{2},...,X^{C^{\prime }}\right]$, $u_{c}\in R^{H\times W}$,$v_{c}^{s}$ is a single-channel kernel in two dimensions, and indicates that $v_{c}$ single chain acts on the corresponding channel X.

(4) $$u_{c}=v_{c}\cdot X=\sum_{s=1}^{c}v_{c}^{s}\cdot x^{s}$$

#### 3.3.3. YOLOv5-SE-BiFPN Detection Model

YOLOv5 is an upgraded version of the YOLO (You Only Look Once) algorithm [37]. According to the depth of the network and the width of the feature map, YOLOv5 includes YOLOv5s, YOLOv5m, YOLOv5l, and YOLOv5x. As the depth and width increase, the number of layers in the network gradually increases, and the structure becomes more complex. To meet the demands of lightweight deployment and real-time detection, reduce the storage space occupied by the model, and improve the recognition speed, this study chooses to take YOLOv5s as the benchmark model and proposes an improved YOLOv5-SE-BiFPN algorithm for detecting the brown spot disease of kidney beans. While ensuring the detection speed, it improves the recognition Precision of brown spot disease in kidney beans.

**Figure 11:**
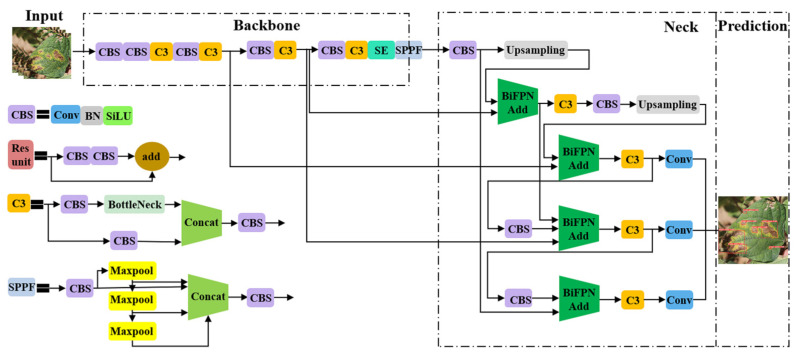
YOLOv5-SE-BiFPN network structure.

The YOLOv5s model mainly consists of four parts: the end of Input, Backbone, Neck, and Prediction [38]. When performing target detection, the model often needs to obtain image feature information from features of different scales and layers to recognize objects of different sizes and shapes. The traditional YOLOv5s model in Backbone and Head uses a simple feature extraction and fusion approach, which makes it difficult to fully utilize feature information from different scales. In this study, the Backbone part of YOLOv5s was adjusted and enhanced. When a leaf with brown spot disease of the kidney bean was input to this model, feature extraction was first performed through the Conv layer, followed by the introduction of the SE module to achieve adaptive learning of the weights of each channel at index nine to optimize the expression of leaf features. Then, at the positions indexed as 13 and 17, the Add operation in BiFPN is used to efficiently fuse the P4 and P3 features in Backbone to enhance the information interaction between the features. Finally, the model gradually transfers and processes the leaf features and outputs the prediction results, including the location and category information of the spots on the leaves of kidney beans. The improved YOLOv5-SE-BiFPN structure is shown in Figure 11.

### 3.4. Training Environment and Evaluation Indicators

The deep learning framework used in this study was PyTorch 1.9.1, and the experiments were run on an Ubuntu-18.04 system using a Xeon(R) Gold 6330-14-core 80 GB RAM CPU, an NVIDIA GeForce RTX 3090 graphics card, and the platform’s built-in CUDA11.1 and cuDNN v8.0.5 Acceleration.

The YOLOv5-SE-BiFPN model hyperparameters are set as follows: the initial learning rate is set to 0.01, and the momentum of the learning rate is set to 0.9. To increase the training speed, the batch size of each training is set to eight times. The resolution size of the input image was set to 640 × 640 pixels based on the common size, and the number of iterations was set to 300.

To evaluate the detection performance of the model, Precision, Recall, and mean average Precision (mAP) are used as evaluation metrics in this study. Among them, Precision denotes the proportion of correctly detected disease frames, Recall denotes the proportion of correctly detected disease prediction frames to all the disease prediction frames present, and mAP is a comprehensive evaluation index of the performance of the target detection model, taking both Precision and Recall into consideration simultaneously, as shown in Equations (5)–(7):

(5) $$\mathrm{Precision}=\frac{\mathrm{TP}}{\mathrm{TP}+\mathrm{FP}}\times 100\%,$$

(6) $$\mathrm{Recall}=\frac{\mathrm{TP}}{\mathrm{TP}+\mathrm{FN}}\times 100\%,$$

(7) $$\mathrm{mAP}=\frac{\sum_{i=1}^{c}\mathrm{AP}\left(c\right)}{C}\%$$

where TP is the number of correctly identified brown patches, FP represents the number of healthy areas incorrectly identified as brown patches, FN is the number incorrectly judged as brown patches, AP is the area under the exact percentage of the checking curve (P–R curve), mAP is the average value of AP for different categories, C is the type of disease, and C = 1, because only a brown patch of kidney bean was to be identified in this experiment.

## 4. Conclusions

This study proposes a YOLOv5-SE-BiFPN-based target detection model for brown spot disease in kidney beans. By introducing the SE module into the Backbone network and applying BiFPN to achieve fast normalized fusion, intra-group channel sharing, bi-directional cross-scale connectivity, and weighted feature fusion, and the number of network parameters is reduced. The provides a reliable solution for the accurate identification of kidney bean brown spot disease.

The experiment results show that the detection Precision of the YOLOv5-SE-BiFPN model for brown spot disease of kidney bean is 94.7%, which is increased by 4.9%, 1.8%, and 3% compared to the YOLOv5, YOLOv5-SE, and YOLOv5-BiFPN models, respectively, and compared to the Faster R-CNN and the EfficientDet models, respectively, improved by 9.4% and 9.5%. Next, the YOLOv5-SE-BiFPN model has a Recall of 88.2%, mAP_@0.5_ is 92.5%, and mAP_@0.5:0.95_ is 69.5%. Compared with the other models, all the indexes of the model showed higher performance, indicating that the improved YOLOv5-SE-BiFPN model has higher accuracy and reliability in the identification of brown spot disease in kidney beans. The results of the ablation experiments conclusively demonstrated the unique advantages and effectiveness of the YOLOv5-SE-BiFPN model in brown spot disease recognition in kidney beans. The test results clearly show that the introduction of the SE module and BiFPN has a significant impact on the recognition of brown spot disease in kidney beans, which not only highlights the contribution of these improved mechanisms but also demonstrates their working mechanism and further enhances the model performance. It provides strong support and validation for the technical improvement of the model.

The YOLOv5-SE-BiFPN model achieved good results in the detection of brown spot disease in kidney beans, but there is still room for improving the detection Precision, and the improved model may still have some limitations in the case of missed or false detection, so it is necessary to further optimize the network structure to improve the network performance of the model. More kidney bean disease data needs to be collected to help the model better understand and distinguish between different types of diseases, to improve its accuracy and robustness in practical applications, and to enable the model to respond to new challenges in kidney bean growth management in a timely and effective manner. 

## Figures and Tables

**Table 1:** Comparison of model performance parameters.

Models	P (%)	R (%)	mAP_@0.5_ (%)	mAP_@[0.5:0.95]_ (%)
YOLOv5	89.8	87.7	66.9	46.6
YOLOv5-SE	92.9	86.7	91.6	68.8
YOLOv5-BiFPN	91.7	85.4	89.7	68.7
YOLOv5-SE-BiFPN	94.7	88.2	92.5	69.5

Notes: P represents Precision; R represents Recall; mAP_@0.5_ represents the mean average Precision at Intersection over Union (IoU) 0.5; and mAP_@[0.5:0.95]_ represents the mean average Precision at different IOU thresholds (from 0.5 to 0.95, step-size 0.05).

**Table 2:** Comparison of model performance parameters.

Models	P (%)	R (%)	mAP_@0.5_ (%)	mAP_@[0.5:0.95]_ (%)
Faster R-CNN	85.3	73.0	82.2	61.6
EfficientDet	85.2	86.3	73.8	53.4
YOLOv5-SE-BiFPN	94.7	88.2	92.5	69.5

Notes: P represents Precision; R represents Recall; mAP_@0.5_ represents the mean average Precision at Intersection over Union (IoU) 0.5; and mAP_@[0.5:0.95]_ represents the mean average Precision at different IOU thresholds (from 0.5 to 0.95, step-size 0.05).

**Table 3:** Comparison of model prediction results.

Models	Accuracy (%)	Rate of Missed and Wrong Detection (%)
YOLOv5	87.07	12.93
YOLOv5-SE	91.68	8.32
YOLOv5-BiFPN	91.14	8.86
YOLOv5-SE-BiFPN	91.84	8.16

## Data Availability

The research project is ongoing, and part of the data is available upon request.
